# Continual measurement of arterial dP/dt_max_ enables minimally invasive monitoring of left ventricular contractility in patients with acute heart failure

**DOI:** 10.1186/s13054-019-2654-8

**Published:** 2019-11-21

**Authors:** Petr Ostadal, Dagmar Vondrakova, Andreas Krüger, Marek Janotka, Jan Naar

**Affiliations:** Cardiovascular Center, Na Homolce Hospital, Roentgenova 2, 150 00 Prague, Czech Republic

**Keywords:** Left ventricle, Contractility, dP/dt, Systemic vascular resistance, Cardiac output, Stroke volume, Acute heart failure

## Abstract

**Background:**

Continuous, reliable evaluation of left ventricular (LV) contractile function in patients with advanced heart failure requiring intensive care remains challenging. Continual monitoring of dP/dt_max_ from the arterial line has recently become available in hemodynamic monitoring. However, the relationship between arterial dP/dt_max_ and LV dP/dt_max_ remains unclear. This study aimed to determine the relationship between arterial dP/dt_max_ and LV dP/dt_max_ assessed using echocardiography in patients with acute heart failure.

**Methods:**

Forty-eight patients (mean age 70.4 years [65% male]) with acute heart failure requiring intensive care and hemodynamic monitoring were recruited. Hemodynamic variables, including arterial dP/dt_max_, were continually monitored using arterial line pressure waveform analysis. LV dP/dt_max_ was assessed using continuous-wave Doppler analysis of mitral regurgitation flow.

**Results:**

Values from continual arterial dP/dt_max_ monitoring were significantly correlated with LV dP/dt_max_ assessed using echocardiography (*r* = 0.70 [95% confidence interval (CI) 0.51–0.82]; *P* < 0.0001). Linear regression analysis revealed that LV dP/dt_max_ = 1.25 × (arterial dP/dt_max_) (*P* < 0.0001). Arterial dP/dt_max_ was also significantly correlated with stroke volume (SV) (*r* = 0.63; *P* < 0.0001) and cardiac output (CO) (*r* = 0.42; *P* = 0.0289). In contrast, arterial dP/dt_max_ was not correlated with SV variation, dynamic arterial elastance, heart rate, systemic vascular resistance (SVR), or mean arterial pressure. Markedly stronger agreement between arterial and LV dP/dt_max_ was observed in subgroups with higher SVR (*N* = 28; *r* = 0.91; *P* <  0.0001), lower CO (*N* = 26; *r* = 0.81; *P* <  0.0001), and lower SV (*N* = 25; *r* = 0.60; *P* = 0.0014). A weak correlation was observed in the subjects with lower SVR (*N* = 20; *r* = 0.61; *P* = 0.0004); in the subgroups with higher CO (*N* = 22) and higher SV (*N* = 23), no significant correlation was found.

**Conclusion:**

Our results suggest that in patients with acute heart failure requiring intensive care with an arterial line, continuous calculation of arterial dP/dt_max_ may be used for monitoring LV contractility, especially in those with higher SVR, lower CO, and lower SV, such as in patients experiencing cardiogenic shock. On the other hand, there was only a weak or no significant correlation in the subgroups with higher CO, higher SV, and lower SVR.

## Background

Left ventricular (LV) contractility is one of the most important parameters determining LV performance and cardiac function and, therefore, directly influences global hemodynamic status [1]. Clinical conditions with impaired LV contractility, such as heart failure or septic cardiomyopathy, are frequent subjects of intensive and acute cardiology care [1]. There is, therefore, an apparent clinical need for bedside measurement or even monitoring of contractility. However, current options for the assessment of LV contractility are significantly limited. The reference method for the measurement of LV contractility (i.e., LV end-systolic elastance [2]) cannot be used in routine clinical practice due to invasiveness and technical issues [3, 4]. The maximum rate of LV pressure rise during ventricular contraction (LV dP/dt_max_) has been adopted as a surrogate marker of LV inotropic state and contractility [5]. This parameter is determinable in clinical settings; however, it requires direct LV pressure measurement, which is impractical and too invasive for LV contractility monitoring. LV dP/dt_max_ can also be estimated non-invasively using echocardiographic techniques [6, 7]. However, although this measurement can be performed repeatedly, it is not feasible for continuous monitoring and is frequently limited by low-quality signal.

Recently, a new surrogate has been proposed—arterial dP/dt_max_. This parameter can be calculated from the arterial pressure waveform, obtained minimally invasively from a peripheral arterial line [8–10] or even non-invasively [11]. Arterial dP/dt_max_ is, therefore, available bedside and in patients with an arterial line already used for pressure monitoring and blood gas analyses; it does not require any additional invasive access. Moreover, arterial dP/dt_max_ can be measured on a beat-by-beat basis and, therefore, continually monitored. On the other hand, arterial dP/dt_max_ is not only determined by LV contraction but is also influenced by various peripheral arterial factors and load conditions [8–11]. Recently, several experimental studies demonstrating a significant relationship between arterial dP/dt_max_ and LV contractility have been published [8–10]. To date, however, clinical studies focusing on the relationship between arterial and LV dP/dt_max_ in patients with acute heart failure requiring intensive care and an arterial line are lacking. The aim of our study was, therefore, to assess the relationship between arterial and LV dP/dt_max_ in this patient population.

## Methods

### Study population

Consecutive patients admitted between January and September 2018 to the cardiology intensive care unit due to acute heart failure requiring an arterial line for invasive blood pressure monitoring and central venous catheter were eligible for the study. Patients with moderate to severe aortic stenosis, those who required mechanical circulatory support, and those with absence of mitral regurgitation enabling measurement of LV dP/dt_max_ were excluded. All patients have to be at the time of measurement on stable doses of inotropes/vasopressors, on stable ventilation support, and with regular cardiac rhythm.

### Hemodynamic measurement

Arterial blood pressure (mean), central venous pressure (mean), heart rate, cardiac output (CO), stroke volume (SV), dynamic arterial elastance, and systemic vascular resistance (SVR) were measured using a clinical monitoring platform (EV1000, equipped with HPI software, Edwards Lifesciences, Irvine, CA, USA) connected to the arterial and central venous lines. SVR was calculated using the formula: SVR = 80 × (MAP − CVP)/CO. The dynamic arterial elastance was defined as the ratio of pulse pressure variation and stroke volume variation. All parameters were calculated in the 20-s interval that contains few respiratory cycles. An arterial catheter was inserted into the left or right radial artery, and the left or right jugular vein was used for central venous access, with the tip of catheter in the superior vena cava.

### dP/dt_max_ measurement

Arterial dP/dt_max_ was measured from the arterial pressure curve by the EV1000 system and HPI software. The system calculates dP/dt_max_ for each beat in a 20-s cycle, then the median value of all the dP/dt_max_ values in the 20-s interval is displayed; values obtained at the time of LV dP/dt_max_ were used in the analysis. LV dP/dt_max_ was measured at the end of expiration using transthoracic echocardiography (Phillips CX50, Amsterdam, The Netherlands) from the analysis of the mitral regurgitation jet by continuous-wave Doppler; calculation was based on the time interval (T) between blood flow of 1 m/s and 3 m/s using the formula: LV dP/dt_max_ = 32/T [6, 7]. Three measurements were performed at one time, and the mean values were used for analysis. The echocardiographic measurements were performed by an experienced physician who was blinded to the arterial dP/dt_max_ values.

### Statistical analysis

Gaussian distribution of the measurement data was tested using the Shapiro-Wilk normality test. Correlation was tested using the Spearman test by calculating the Spearman correlation coefficient. LV and arterial dP/dt_max_ were compared using the Bland-Altman analysis. Linear regression was used to derive the equation representing the relationship between LV and arterial dP/dt_max_. The analyses were performed using GraphPad Prism version 7 (GraphPad Software, Inc., La Jolla, CA, USA) and MedCalc (MedCalc Software, Ostend, Belgium); *P* < 0.05 was considered to be statistically significant.

## Results

Forty-eight patients were enrolled in the study; baseline characteristics of the study population are summarized in Table 1. The mean age was 70.4 years, the majority were males (65%), and the main cause of acute heart failure was ischemic cardiomyopathy (65%). Eighty-five percent of patients were treated with intravenous inotropes, and the majority required vasopressors (73%).

**Table 1:** Baseline characteristics (*n* = 48)

Characteristics	Value
Male sex	31 (65)
Age (years, mean ± SD)	70.4 ± 8.1
Decompensated CHF	31 (65)
De novo AHF	17 (35)
Ischemic cardiomyopathy	31 (65)
Dilated cardiomyopathy	7 (15)
Acute myocardial infarction	15 (31)
Severe mitral regurgitation	19 (40)
Left ventricular ejection fraction (%, mean ± SD)	28 ± 10
Mechanical ventilation	17 (35)
Inotropes (dobutamine, milrinone)	41 (85)
Vasopressors (norepinphrine, vasopressin)	35 (73)
Heart rate (beats/min, mean ± SD)	92.7 ± 11.7
Mean arterial pressure (mmHg, mean ± SD)	74.0 ± 5.2
Cardiac output (L/min, mean ± SD)	6.0 ± 1.2
Stroke volume (mL, mean ± SD)	65.5 ± 10.1
Systemic vascular resistance (dyn·s/cm^5^, mean ± SD)	891.5 ± 236.3

Data presented as *n* (%) unless otherwise indicated. *CHF* chronic heart failure, *AHF* acute heart failure

**Fig. 1:**
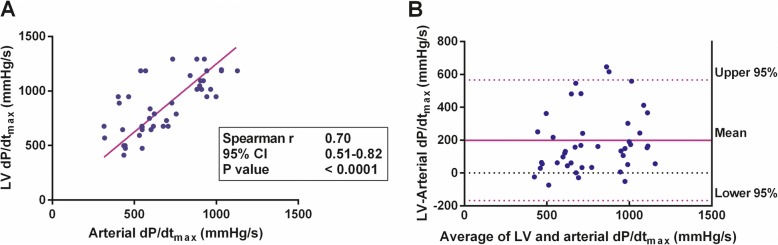
Arterial and left ventricular (LV) dP/dt_max_. **a** Correlation between arterial and LV dP/dt_max_ with linear regression curve. **b** The Bland-Altman plot of agreement between arterial and LV dP/dt_max_

The values from continual arterial dP/dt_max_ monitoring were significantly correlated with LV dP/dt_max_ assessed using echocardiography (*r* = 0.70 [95% confidence interval (CI) 0.51–0.82]; *P* < 0.0001) (Fig. 1). Linear regression revealed that LV dP/dt_max_ = 1.25 × (arterial dP/dt_max_) (*P* < 0.0001). Arterial dP/dt_max_ was significantly correlated with SV (*r* = 0.63 [95% CI 0.41–0.78]; *P* < 0.0001) and CO (*r* = 0.42 [95% CI 0.14–0.63]; *P* = 0.003). In contrast, arterial dP/dt_max_ was not correlated with SV variation, dynamic arterial elastance, heart rate, SVR, or mean arterial pressure (Table 2).

**Table 2:** Correlation between arterial dP/dt_max_ and other recorded hemodynamic variables

Variable	Spearman’s *r*	95% confidence interval	*P* value
Stroke volume	0.6297	0.4137 to 0.7786	< 0.0001
Cardiac output	0.419	0.1446 to 0.6336	0.003
Stroke volume variation	− 0.2635	− 0.5159 to 0.03099	0.0703
Dynamic arterial elastance	0.06733	− 0.2293 to 0.3525	0.6493
Heart rate	− 0.06501	− 0.3505 to 0.2315	0.6607
Systemic vascular resistance	− 0.1734	− 0.4431 to 0.1251	0.2385
Mean blood pressure	0.345	− 0.02172 to 0.6298	0.0574

The correlation between arterial and LV dP/dt_max_ was calculated also in subgroups above and below the mean value of the recorded variables. Marked differences in the correlation between arterial and LV dP/dt_max_ were observed in the subgroups based on the mean SVR, CO, and SV; on the other hand, similar correlation was observed in the subgroups based on SV variation (*r* = 0.54 vs. *r* = 0.59), dynamic arterial elastance (*r* = 0.70 vs. *r* = 0.73), heart rate (0.68 vs. 0.72), and mean arterial pressure (*r* = 0.80 vs. *r* = 0.76).

### SVR subgroups

**Fig. 2:**
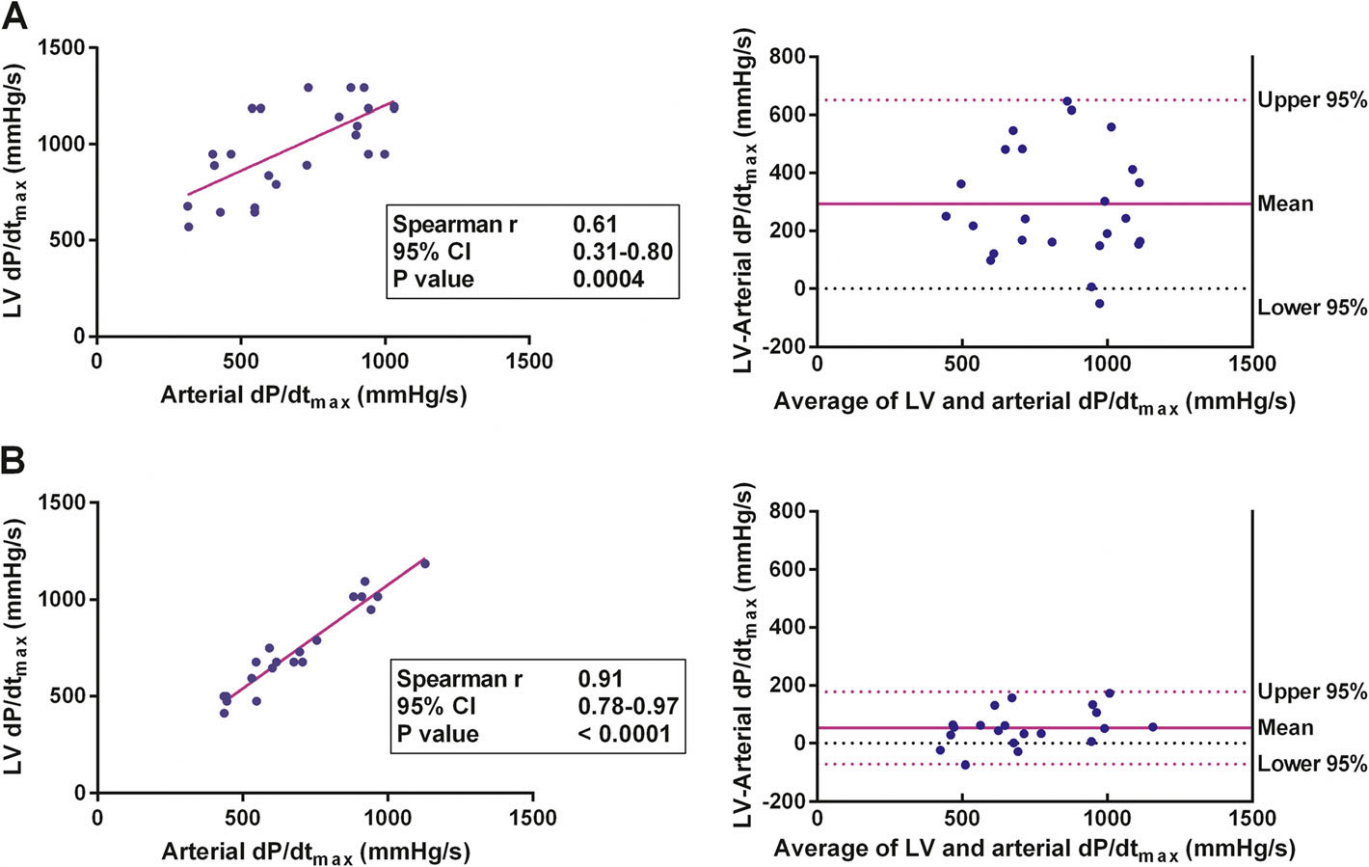
Arterial and left ventricular (LV) dP/dt_max_ in subgroups according to systemic vascular resistance (SVR). **a** Subgroup with lower SVR (< 900 dyn·s/cm^5^, *N* = 20)—correlation between arterial and LV dP/dt_max_ with linear regression curve (left), and the Bland-Altman plot of the agreement between arterial and LV dP/dt_max_ (right). **b** Subgroup with higher SVR (> 900 dyn·s/cm^5^, *N* = 28)—correlation between arterial and LV dP/dt_max_ with linear regression curve (left), and the Bland-Altman plot of the agreement between arterial and LV dP/dt_max_ (right)

The study population was divided into two groups according to the mean level of SVR (> or < 900 dyn·s/cm^5^). In the subgroup of patients with lower SVR (< 900 dyn·s/cm^5^ [*n* = 28]), a statistically significant correlation between arterial dP/dt_max_ and LV dP/dt_max_ was found (*r* = 0.61 [95% CI 0.31 to 0.80]; *P* = 0.0004). However, in the subgroup with higher SVR (> 900 dyn·s/cm^5^ [*n* = 20]), the correlation between arterial and LV dP/dt_max_ was markedly stronger and highly statistically significant (*r* = 0.91 [95% CI 0.78 to 0.97]; *P* < 0.0001) (Fig. 2). Linear regression analysis revealed that in subgroup with higher SVR, LV dP/dt_max_ could be calculated according to the equation: LV dP/dt_max_ = 1.08 × (arterial dP/dt_max_).

### CO subgroups

**Fig. 3:**
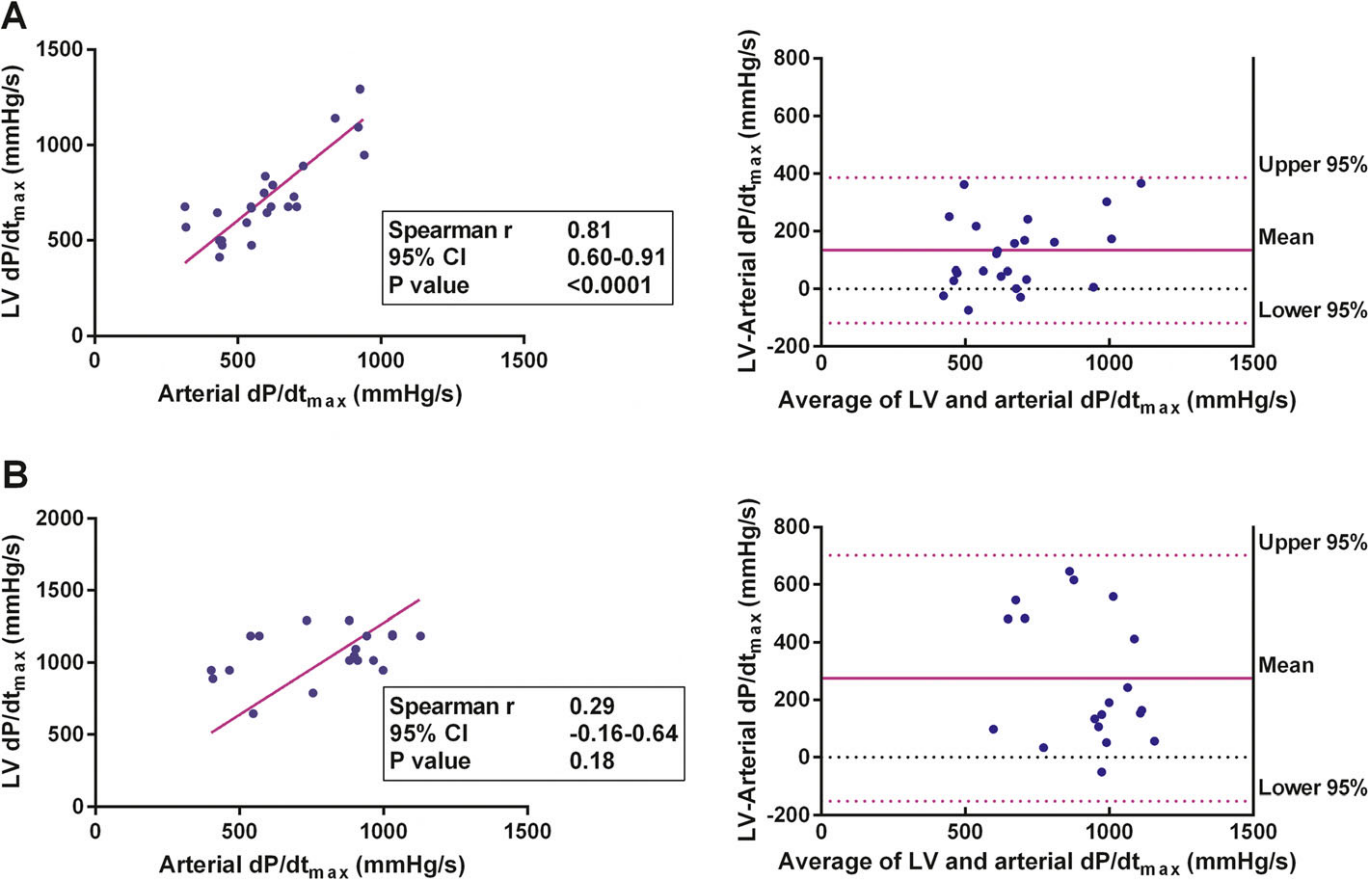
Arterial and left ventricular (LV) dP/dt_max_ in subgroups according to cardiac output (CO). **a** Subgroup with lower CO (< 6 L/min, *N* = 26)—correlation between arterial and LV dP/dt_max_ with linear regression curve (left), and the Bland-Altman plot of the agreement between arterial and LV dP/dt_max_ (right). **b** Subgroup with higher CO (> 6 L/min, *N* = 22)—correlation between arterial and LV dP/dt_max_ with linear regression curve (left), and the Bland-Altman plot of the agreement between arterial and LV dP/dt_max_ (right)

In the subgroup of patients with lower CO (< 6 L/min [*n* = 26]), a strong and highly statistically significant correlation between arterial dP/dt_max_ and LV dP/dt_max_ was found (*r* = 0.81 [95% CI 0.60 to 0.91]; *P* < 0.0001). In contrast, in the subgroup with higher CO (> 6 L/min [*n* = 22]), the correlation between arterial and LV dP/dt_max_ was not statistically significant (*r* = 0.29 [95% CI − 0.16 to 0.64]; *P* = 0.18) (Fig. 3). Linear regression revealed that in the subgroup with lower CO, LV dP/dt_max_ could be calculated according to the equation: LV dP/dt_max_ = 1.21 × (arterial dP/dt_max_).

### SV subgroups

**Fig. 4:**
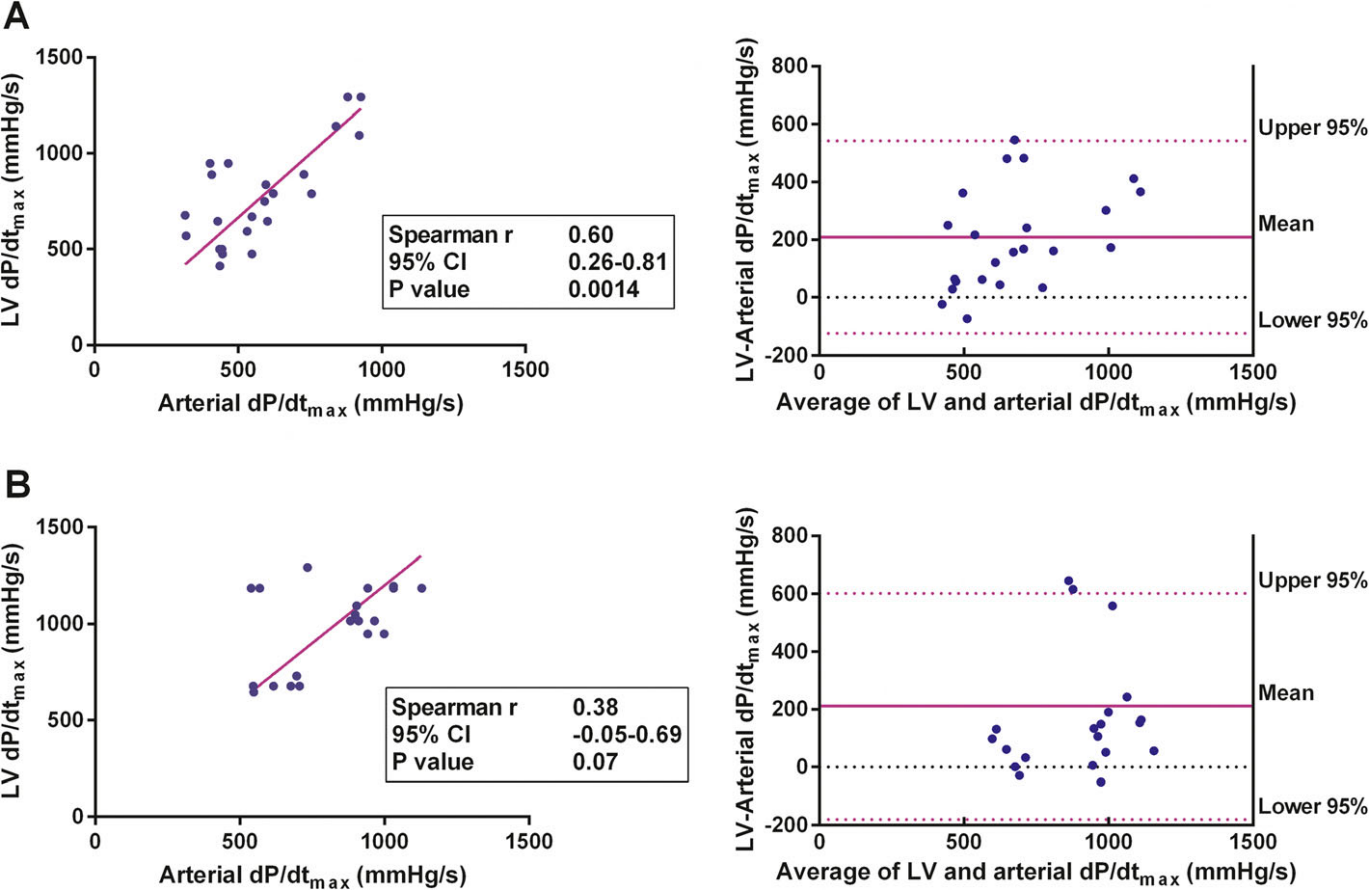
Arterial and left ventricular (LV) dP/dt_max_ in subgroups according to stroke volume (SV). **a** Subgroup with lower SV (< 65 mL, *N* = 25)—correlation between arterial and LV dP/dt_max_ with linear regression curve (left), and the Bland-Altman plot of the agreement between arterial and LV dP/dt_max_ (right). **b** Subgroup with higher SV (> 65 mL, *N* = 23)—correlation between arterial and LV dP/dt_max_ with linear regression curve (left), and the Bland-Altman plot of the agreement between arterial and LV dP/dt_max_ (right)

In the subgroup of patients with lower SV (< 65 mL [*n* = 25]), a statistically significant correlation between arterial dP/dt_max_ and LV dP/dt_max_ was found (*r* = 0.60 [95% CI 0.26 to 0.81]; *P* = 0.0014). In contrast, in the subgroup with higher SV (> 65 mL [*n* = 23]), the correlation of between arterial and LV dP/dt_max_ was not statistically significant (*r* = 0.38 [95% CI − 0.05 to 0.69]; *P* < 0.07) (Fig. 4). Linear regression revealed that in the subgroup with lower SV, LV dP/dt_max_ could be calculated according to the equation: LV dP/dt_max_ = 1.33 × (arterial dP/dt_max_).

## Discussion

Our results demonstrate that in adult patients with acute heart failure, the values of arterial dP/dt_max_, which can be continuously monitored by analysis of the pressure waveform, were significantly correlated with LV dP/dt_max_. An even better agreement between arterial dP/dt_max_ and LV dP/dt_max_ was observed in subgroups with higher SVR, lower CO, and lower SV. This observation is particularly important because monitoring LV contractility is most desirable in patients with heart failure with critical hemodynamic collapse, such as in cardiogenic shock, characterized by increased SVR and decreased CO and SV. In contrast, in the subgroups with lower SVR, higher CO, and higher SV, the correlation was weak or even absent.

Acute heart failure is a critical condition in which LV contractility is commonly depressed, and therapeutic strategies are frequently based on increasing inotropy [12]. Although LV contractility status can be routinely and intermittently assessed using echocardiography in patients with acute heart failure, even with the use of new techniques, the measurement can be inaccurate and interpretation difficult [13–17]. There are currently highly limited options for continuous LV contractility monitoring. In contrast to echocardiographic techniques, the maximal rate of arterial pressure increases during systole (arterial dP/dt_max_) can be easily and continuously calculated from the pressure waveform. It has been shown in animal studies that arterial dP/dt_max_ may correlate with LV contractility status under various hemodynamic conditions. In a porcine model of endotoxin-induced shock and catecholamine infusion, Morimont et al. [8] observed that arterial dP/dt_max_ was significantly correlated with LV contractility measured by LV end-systolic elastance (Ees) or LV dP/dt_max_. The authors also found a better correlation when adequate vascular filling according to the arterial pulse pressure variation was achieved. These results are consistent with our observations. We have demonstrated a strong relationship between arterial and LV dP/dt_max_ under higher SVR, which could be also a result of increased vascular filling. Monge Garcia et al. [10] analyzed the relationship among arterial dP/dt_max_, LV dP/dt_max_, and Ees in sequential changes of afterload, preload, and contractility in pigs. In this study, arterial dP/dt_max_ enabled the tracking of Ees changes, especially during the modification of afterload and contractility, and changes in cardiac contractility (i.e., Ees) were the main determinants of arterial dP/dt_max_ changes. Moreover, these observations are in good agreement with our results; Monge Garcia et al. recorded higher values of LV dP/dt_max_ in comparison with arterial dP/dt_max_, similar to our study. A good correlation between arterial dP/dt_max_ and LV dP/dt_max_ in heart failure patients was also reported in the study by Tartiere et al. [11], in which the dP/dt_max_ from the radial artery was assessed non-invasively using applanation tonometry and LV dP/dt_max_. Our results are also consistent with the observation by Scolletta et al. [18] who reported significant correlation between arterial dP/dt_max_ and LV dP/dt_max_ in a group of critically ill patients. Furthermore, and again similar to our results, a very close linear relationship was found between arterial dP/dt_max_ from the femoral artery and invasively measured LV dP/dt_max_ (with a catheter in the LV) in patients scheduled for coronary artery bypass surgery [9]; arterial dP/dt_max_ also underestimated LV dP/dt_max_ in this study.

In contrast, Kim et al. [19] studied the relationship between arterial dP/dt_max_ from the radial artery, aortic dP/dt_max_, and selected echocardiographic variables such as LV ejection fraction or LV fractional shortening in children undergoing congenital heart disease surgery. They did not find a significant correlation between arterial dP/dt_max_ and the other variables, and these observations were explained by differences between radial artery and aortic pressure waveforms. The discrepancy with our results can be elucidated by the other measured parameters in Kim et al.’s study. While we used LV dP/dt_max_ as a surrogate parameter of LV contractility, Kim et al. measured LV ejection fraction and LV fractional shortening, which are not the accepted markers of LV contractility, because they are influenced by many other factors (e.g., preload, mitral regurgitation). The observations in the study by Kim et al. could be also influenced by CO or SVR (or vascular filling), which were not recorded. Recently, Vaquer et al. [20] published a study focused on femoral arterial dP/dt_max_ in critically ill patients, predominantly with septic shock. They observed increase in arterial dP/dt_max_ after administration of dobutamine and norepinephrine but not after volume expansion. The changes in arterial dP/dt_max_ were strongly correlated with the changes in pulse pressure and systolic arterial pressure in all interventions including volume expansion. Vaquer et al. conclude that femoral arterial dP/dt_max_ is, therefore, an unreliable estimate of LV systolic function [20]. Our study was not designed to evaluate the relationship between dP/dt_max_ and LV systolic function; we focused on the comparison of the arterial dP/dt_max_ and the LV dP/dt_max_, as a surrogate marker of LV contractility. Although the study group characteristics are different, the observation of increase in arterial dP/dt_max_ after administration of agents with inotropic effect (dobutamine, norepinephrine) in the study by Vaquer et al. [20] is in a good agreement with our results showing significant correlation between arterial and LV dP/dt_max_. Our observation that correlation between arterial and LV dP/dt_max_ depends on SVR is also consistent with the results by Vaquer et al. [20] describing that femoral dP/dt_max_ is influenced by LV preload and afterload.

Several authors reported that the arterial dP/dt_max_ is significantly influenced by vascular filling conditions [8, 10, 20]. We observed in our study that the correlation between arterial and LV dP/dt_max_ was influenced by SVR, which reflects loading conditions; however, SV variation or dynamic arterial elastance had no effect on the relationship between arterial and LV dP/dt_max_. This contrast can be at least partly explained by the fact that in our study, the measurement was done in a single time point, while in the other studies, serial measurements were performed enabling evaluation of dynamic changes. In addition, we included entirely patients with acute left heart failure, where the LV preload is usually increased that may or may not be accompanied by changes in other indirect markers of vascular filling. Our study had several limitations, the first of which was possible bias caused by the small sample size. We designed only a pilot study focusing primarily on feasibility; however, a larger trial should be performed to confirm our results. We also acknowledge that arterial dP/dt_max_ is not only a function of LV contractility but is influenced by many other factors, at least by arterial vessel wall characteristics (e.g., arterial elasticity and stiffness), which was not assessed in the present study. We did not record pulse pressure enabling to calculate arterial elastance. It can be assumed that there can be marked individual differences in arterial system properties in heart failure patients, who often present with other diseases and various degrees of peripheral atherosclerosis. Patients with moderate to severe aortic stenosis were ineligible for this study; however, we cannot exclude the possibility that even mild aortic stenosis may have influenced the results. Moreover, in our study, we have performed only single measurements at one time point in each patient. Our study, therefore, was not designed to evaluate the trends in arterial dP/dt_max_ changes. In addition, while the LV dP/dt_max_ values were obtained at the end of expiration, the arterial dP/dt_max_ were calculated as a median value from 20-s interval, therefore not at the same period of respiratory cycle. Finally, LV dP/dt_max_ is only a surrogate marker of LV contractility and measurement of this parameter using echocardiography can be inaccurate.

## Conclusions

Our results suggest that in patients with acute heart failure requiring intensive care with an arterial line, continuous calculation of arterial dP/dt_max_ may be used for monitoring LV contractility, especially in those with higher SVR, lower CO, and lower SV, such as in patients experiencing cardiogenic shock. On the other hand, there was only a weak or no significant correlation in the subgroups with higher CO, higher SV, and lower SVR.

## Data Availability

The datasets used and/or analyzed during the current study are available from the corresponding author on reasonable request.

## Abbreviations

CO: Cardiac output
Ees: End-systolic elastance
LV: Left ventricular
SV: Stroke volume
SVR: Systemic vascular resistance
T: Time interval
